# A Comparative Transcriptomic Analysis of X-Ray Irradiation Responses Identifies Candidate Biomarker Genes in *Bactrocera dorsalis* Larvae

**DOI:** 10.3390/insects17080804

**Published:** 2026-08-03

**Authors:** Baishu Li, Sihan Jin, Haomiao Li, Rui Li, Li Li, Junzheng Zhang, Tao Liu

**Affiliations:** 1 Chinese Academy of Quality and Inspection & Testing, Beijing 100123, China; libaishu@163.com; 2 State Key Laboratory of Agricultural and Forestry Biosecurity, MARA Key Lab of Surveillance and Management for Plant Quarantine Pests, College of Plant Protection, China Agricultural University, Beijing 100193, China; jinsihan@cau.edu.cn (S.J.); 2017319010117@cau.edu.cn (H.L.); lirui@cau.edu.cn (R.L.)

**Keywords:** *Bactrocera dorsalis*, X-ray irradiation, phytosanitary, transcriptome, biomarker

## Abstract

*Bactrocera dorsalis* (Diptera: Tephritidae) ranks among the most serious quarantine pests threatening global fruit and crop production. Irradiation has been adopted worldwide as an eco-friendly phytosanitary treatment strategy, yet how *B. dorsalis* responds to irradiation is not fully understood. To investigate the transcriptional response to irradiation, third-instar *B. dorsalis* larvae were exposed to two X-ray doses: a sub-effective dose of 15 Gy and a phytosanitary dose of 120 Gy. Comparative transcriptomic profiling revealed a clear dose-dependent escalation in the number of differentially expressed gene (DEGs). While 15 Gy primarily disrupted basic metabolism and cuticle integrity, 120 Gy induced a systemic metabolic collapse coupled with severe genotoxic stress, activating DNA repair, lysosomal degradation, and the JAK-STAT signaling pathway. Notably, we identified nine dose-specific candidate biomarker genes that are exclusively regulated at the phytosanitary dose. These findings elucidate the molecular network mediating radiation-induced mortality and provide rapid molecular diagnostic tools for validating phytosanitary irradiation efficacy and optimizing pest management strategies.

## Simple Summary

The oriental fruit fly (*Bactrocera dorsalis*) is a major global agricultural pest, and X-ray irradiation is a key eco-friendly phytosanitary method. In this study, we exposed *B. dorsalis* larvae to a sub-effective dose (15 Gy) and a fully effective phytosanitary dose (120 Gy) of X-rays to understand how the insects respond at a molecular level. We discovered that the lethal 120 Gy dose triggers a severe biological collapse, shutting down essential metabolism while activating intense cellular stress and DNA repair pathways, such as the JAK-STAT signaling cascade. Most importantly, we identified nine biomarkers that only react to the lethal 120 Gy dose. These markers can be used to quickly and accurately verify whether irradiation treatments have successfully met quarantine safety standards, offering a faster alternative to time-consuming biological testing methods.

## 1. Introduction

The oriental fruit fly, *Bactrocera dorsalis* (Hendel), is a significant agricultural pest that poses severe threats to fruit and vegetable production worldwide [1]. *B. dorsalis* has spread to many countries in Asia, Africa, and the Americas, leading to considerable economic losses [2]. *B. dorsalis* infests a wide range of host plants, particularly tropical and subtropical fruits. Female flies deposit eggs within the fruit, and the developing larvae cause substantial damage to fruits [1,2]. Managing the oriental fruit fly is challenging due to its broad host range, high fecundity, and environmental adaptability. While chemical insecticides are the primary control method, widespread resistance has emerged in many regions [3,4,5,6]. Therefore, phytosanitary treatments are recognized as essential measurements that serve as the first line of defense against *B. dorsalis* invasion [7].

Current phytosanitary methods for managing *B. dorsalis* include heat treatment, cold treatment, ionizing radiation, and methyl bromide fumigation [7]. Irradiation is a safe and effective approach for meeting quarantine requirements while minimizing postharvest losses [8]. The global shift toward ionizing radiation is driven by several key factors, notably the high tolerance of fresh produce to irradiation compared to conventional treatments [9]. Furthermore, increasing restrictions on chemical fumigants, growing international acceptance of irradiation protocols, and the technology’s suitability for diverse commodities have accelerated its adoption. As a result, the use of ionizing radiation for both domestic and international shipments is expected to expand significantly [10].

Phytosanitary irradiation uses high-frequency electromagnetic waves (e.g., X-rays and gamma rays) or high-energy particles like electron beams. Despite their different physical forms, their energy transfer mechanisms and impacts on insect pests are broadly similar [9]. Ionizing radiation can directly strip electrons from critical biological molecules such as DNA and cause irreversible damage. Radiation also generates reactive species like free electrons, ions, and free radicals that subsequently oxidize and damage various biomolecules. In either scenario, the structural integrity and activity of essential cellular components are severely compromised [10]. Such molecular damage causes cellular dysfunction and impairs normal insect development, effectively preventing adult emergence in infested commodities.

The cellular and molecular responses to irradiation are extensively investigated in the model insect *Drosophila melanogaster* [11]. Irradiation triggers widespread and persistent oxidative stress in larval cells, leading to severe DNA damage. Consequently, the activation of DNA repair genes is essential for irradiation tolerance [12]. Beyond DNA damage, oxidative stress also disrupts other biomolecules and cellular organelles. Insects counteract these stresses through responsive pathways, including antioxidant defense systems, molecular chaperones, detoxification enzymes, and ATP-binding cassette (ABC) transporters. ABC transporters are highly conserved across insects and play crucial roles in stress responses and physiological homeostasis [13,14]. Furthermore, the JAK-STAT signaling pathway serves as an evolutionarily conserved mechanism against irradiation-induced stress [15,16]. However, the involvement of these specific pathways in the irradiation response of *B. dorsalis* and other tephritid fruit flies remains largely unexplored.

The efficacy of irradiation against tephritid fruit flies, including *B. dorsalis*, has been well documented [17,18,19,20,21,22,23]. Effective doses typically range from 50 to 400 Gy, depending on the pest species. Specifically, the International Plant Protection Convention (IPPC) recommends a minimum absorbed dose of 116 Gy to prevent the emergence of *B. dorsalis* adults [24]. However, several major importing countries currently do not accept phytosanitary irradiation [25]. There is a growing emphasis on optimizing irradiation doses and broadening their applicability to enhance the implementation of phytosanitary irradiation. A deeper understanding of the biological and molecular responses of *B. dorsalis* to varying irradiation doses would aid in the development of essential tools for evaluating treatment efficacy and promoting sustainable management practices.

## 2. Materials and Methods

### 2.1. Insect Rearing

The *B. dorsalis* stock was collected from a mango orchard in the Guangxi Zhuang Autonomous Region, China, and maintained in the laboratory of the Chinese Academy of Quality and Inspection and Testing (CAQIT). The adult diet consisted of sucrose and yeast extract (mixed at 3:1). Larvae were cultivated on an artificial diet formulated with sucrose, wheat bran, beer yeast, sorbic acid, ascorbic acid, methylparaben, and water. The rearing environment was maintained at 25 ± 2 °C, 70 ± 5% relative humidity, and a 12:12 h dark-to-light photoperiod [26]. All irradiation treatments used third-instar larvae sourced from the third to sixth generations.

### 2.2. Irradiation Treatments

An RS-2000 ProX irradiator (Rad Source-Technologies, Inc., Coral Springs, FL, USA) at the CAQIT Irradiation Processing Laboratory was used for X-ray irradiation under 220 kV and 17.6 mA conditions. The test larvae were sealed in self-sealing bags and placed in a 17 × 15 × 17-inch exposure chamber. A RadCal dosimeter (model 2086, RadCal Corp., Monrovia, CA, USA) with a 10 × 6^−0.6^ ion chamber was used to record the cumulative dose. Radiation was delivered at 9.0 Gy/min under ambient conditions, terminating at the target dose [26]. Irradiation treatments were administered by qualified personnel at the CAQIT Irradiation Processing Laboratory in strict compliance with institutional guidelines.

### 2.3. Developmental Test

Upon completion of the treatments, 150 late third-instar larvae were randomly collected from every group, encompassing both control and all treatment groups. Each larva was individually placed in a plastic container (5 cm diameter, 5 cm height) filled with sterile moist sand within an environmental control chamber (KBF 720; WTC Binder, Tuttlingen, Germany). The rearing parameters were fixed at 25 °C, 60% RH, and a 12:12 h dark-to-light photoperiod. Individual developmental progression was monitored and documented daily at 9:00 a.m. for 14 consecutive days [26].

### 2.4. Transcriptome Analysis

Third-instar *B. dorsalis* larvae were irradiated with 15 Gy and 120 Gy doses. Each treatment included three biological replicates, with 50 larvae per replicate. Immediately after irradiation, the larvae were washed with double-distilled water, blotted dry with filter paper, transferred into sterilized 1.5 mL centrifuge tubes at a density of 10 larvae per tube, flash-frozen in liquid nitrogen, and sent to Beijing Biomics Biotechnology (Beijing, China) for transcriptome sequencing. Total RNAs were extracted using TRIzol reagent (15596018CN, ThermoFisher, Waltham, MA, USA). RNA quality was evaluated before library construction. RNA purity and concentration were assessed using a NanoDrop spectrophotometer, and RNA integrity was examined using an Agilent 2100 Bioanalyzer. After quality control, mRNAs were enriched using oligo (dT) magnetic beads and then fragmented into short fragments. First-strand cDNA was synthesized using random hexamer primers, followed by second-strand cDNA synthesis. The double-stranded cDNA fragments were purified, end-repaired, A-tailed, ligated to sequencing adapters, size-selected, and amplified by PCR to generate the final cDNA libraries. Library quality was evaluated using Qubit 2.0, Agilent 2100, and qPCR. Qualified libraries were sequenced on an Illumina HiSeq platform.

Raw reads were filtered to obtain clean reads by removing adaptor-containing reads, reads with more than 10% ambiguous bases, and low-quality reads in which bases with a quality score ≤ 10 accounted for more than 50% of the total read length. The clean reads were then mapped to the reference genome of *B. dorsalis* using HISAT2. The mapped reads were used for library quality assessment, transcript assembly, gene expression quantification, and downstream differential expression analysis. Transcript assembly and novel transcript identification were performed using StringTie, and the assembled transcripts were compared with the reference genome annotation.

Gene expression levels were quantified using htseq-count and normalized as FPKM values. Pearson correlation analysis and hierarchical clustering were performed based on the FPKM values of all samples to evaluate the reproducibility among biological replicates. Pearson correlation coefficients among samples were calculated using the cor function in R, and hierarchical clustering analysis was conducted based on the correlation matrix. Differential expression analysis was conducted using DESeq2. Genes with |Fold Change| ≥ 2 and FDR < 0.01 were considered differentially expressed genes (DEGs). The FDR was calculated by adjusting the original *p* values using the Benjamini–Hochberg method. GO enrichment analysis and KEGG pathway enrichment analysis were then performed to identify the biological processes and pathways significantly affected by irradiation.

### 2.5. K-Means Clustering of Gene Expression Profiles

To investigate the global transcriptional response patterns of *B. dorsalis* larvae under different irradiation doses, gene expression pattern clustering analysis was performed using all expressed genes detected in the transcriptome dataset. The FPKM values of each gene across the control, 15 Gy, and 120 Gy groups were transformed and standardized to allow comparison of relative expression trends among treatments. Genes with similar expression trajectories across the three treatment groups were clustered using K-means clustering. The optimal number of clusters was evaluated by comparing adjusted R-squared and delta R-squared values across different cluster numbers. Genes within the same cluster were considered to share similar transcriptional response patterns, and the average expression trend of each cluster was used to represent the major expression pattern of the corresponding gene set. Functional interpretation of the major clusters was performed according to their expression trends and enriched biological functions.

## 3. Results

### 3.1. X-Ray Irradiation Induces Prominent Changes in Gene Expression

**Figure 1:**
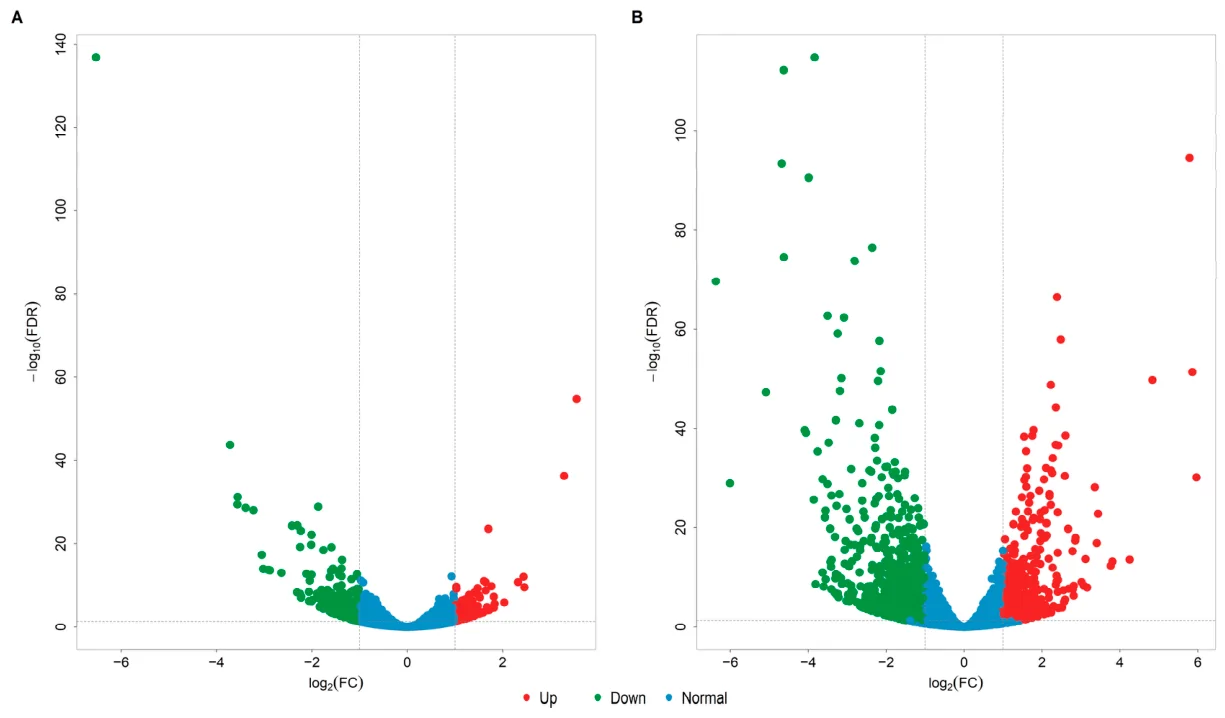
Volcano plot analysis of differentially expressed genes in third-instar *B. dorsalis* larvae following X-ray irradiation. (**A**) Transcriptional response to a sub-effective 15 Gy dose compared to the untreated control group. (**B**) Transcriptional response to a phytosanitary 120 Gy dose compared to the untreated control group. In both panels, each individual dot represents a single gene. The *x*-axis represents the magnitude of expression change as the log_2_ fold change (log_2_FC), while the *y*-axis represents the statistical significance as the negative base-10 logarithm of the false discovery rate (−log_10_ FDR). Significantly up-regulated genes (meeting the threshold of log_2_FC ≥ 1 and FDR < 0.01) are highlighted in red, and significantly down-regulated genes (log_2_FC ≤ −1 and FDR < 0.01) are highlighted in green.

X-ray irradiation is effective in a dose-dependent manner, but the molecular response of *B. dorsalis* larvae to irradiation remains poorly understood. We used mRNA sequencing to examine the effects of X-ray irradiation on larval gene expression. Two doses were selected for this study. Developmental tests confirmed complete inhibition of adult emergence at 120 Gy, whereas the 15 Gy dose had no significant effect, consistent with our previous findings [26]. Pearson correlation analysis based on FPKM values showed high consistency among biological replicates (r > 0.90), indicating good reproducibility and reliability of the RNA-seq data (Figure S1A). Hierarchical clustering further revealed clear separation among different treatment groups, suggesting that the treatments induced distinct transcriptional responses (Figure S1B). A total of 230 DEGs were identified in larvae that received 15 Gy irradiation (Table S1), including 147 down-regulated genes and 83 up-regulated genes (Figure 1A). Exposure to the higher dose impacted significantly more genes. A total of 978 genes were altered after 120 Gy irradiation (Table S2), with 615 down-regulated and 363 up-regulated (Figure 1B). These results demonstrate a clear dose-dependent impact of the *B. dorsalis* transcriptome. The higher proportion of down-regulated genes at both doses suggests an overall inhibitory effect on transcriptional activity.

### 3.2. Signature of Low-Dose X-Ray Irradiation-Induced Gene Expression Changes

**Figure 2:**
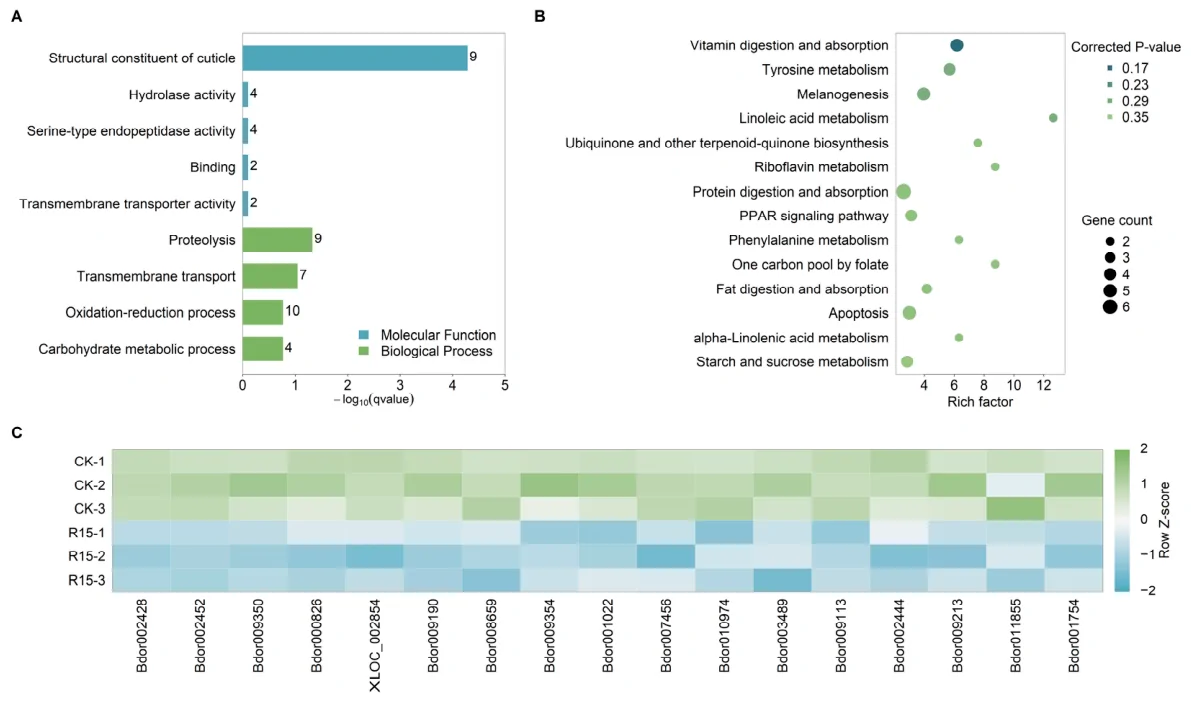
Analysis of transcriptomic response to 15 Gy irradiation in third-instar larvae. (**A**) Gene Ontology (GO) enrichment analysis of Biological Process (green bars) and Molecular Function (blue bars) terms affected by irradiation. The *y*-axis lists the specific enriched terms, while the *x*-axis represents the enrichment significance level (−log_10_ q-value). The numerical value at the end of each bar indicates the exact count of DEGs assigned to that specific term. (**B**) Kyoto Encyclopedia of Genes and Genomes (KEGG) pathway enrichment analysis represented as a bubble plot. The *y*-axis lists the affected pathways, and the *x*-axis represents the Rich factor (defined as the proportion of DEGs mapped to a given pathway relative to the total number of annotated genes in that pathway). The size of each bubble corresponds to the total number of DEGs associated with the pathway, and the color intensity represents the corrected *p*-value (false discovery rate), with darker shades indicating higher statistical significance. (**C**) Heatmap displaying the expression profiles of genes related to insect cuticle structure. Rows represent individual biological replicates from the control (CK) and 15 Gy treatment (R15) groups, while columns represent individual gene symbols (listed at the bottom). The color scale indicates standardized relative expression levels, ranging from blue (low expression, Z-score = −2) to green (high expression, Z-score = 2).

To define the molecular signature of low-dose radiation stress, we profiled the transcriptomic changes induced by a 15 Gy exposure. GO enrichment analysis identified several key biological processes perturbed by this treatment. These included cuticle structural components, proteolytic activity, transmembrane transport, redox processes, and carbohydrate metabolism (Figure 2A). KEGG pathway analysis revealed significant disruption to metabolic activities. Affected pathways included nutrient digestion and absorption (vitamins, proteins, and lipids) and the metabolism of specific compounds such as tyrosine, starch and sucrose, riboflavin, phenylalanine, and linoleic acid (Figure 2B). Cuticle-related pathways were particularly sensitive to this treatment. Specifically, 17 of the 155 down-regulated genes encode proteins essential for cuticle formation and maintenance (Figure 2C). This indicates that sub-lethal radiation doses impair structural integrity during larval development. Additionally, the enrichment of apoptosis-related genes indicates that 15 Gy irradiation activates programmed cell death. Overall, these results demonstrate that low-dose X-ray irradiation disrupts both general nutrient metabolism and specific biochemical processes.

### 3.3. Signature of High-Dose X-Ray Irradiation-Induced Gene Expression Changes

**Figure 3:**
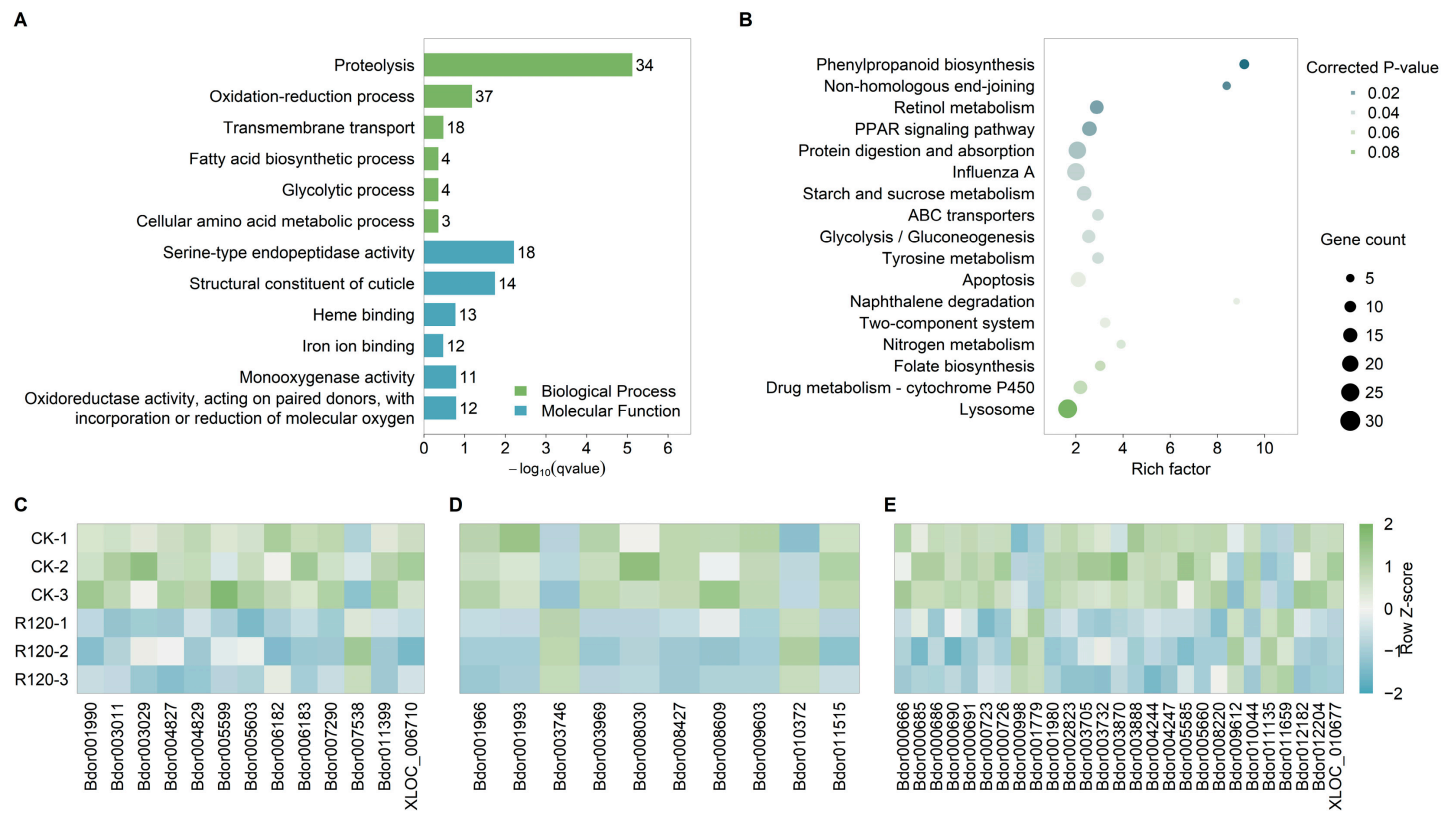
Transcriptomic response of third-instar *B. dorsalis* larvae to a phytosanitary 120 Gy X-ray irradiation. (**A**) GO enrichment analysis of biological processes and molecular functions affected by irradiation. Biological Process terms are represented by green bars, and Molecular Function terms by blue bars. The *y*-axis lists the specific enriched terms, while the *x*-axis represents the enrichment significance level (−log_10_ q-value). The numerical value at the end of each bar indicates the exact count of DEGs assigned to that term. (**B**) KEGG pathway enrichment analysis, represented as a bubble plot. The *y*-axis lists the affected pathways. The *x*-axis represents the Rich factor (defined as the proportion of DEGs mapped to a given pathway relative to the total number of annotated genes in that pathway). Bubble size corresponds to the total number of DEGs associated with the pathway. Color intensity indicates the corrected *p*-value (false discovery rate), with darker shades reflecting higher statistical significance. (**C**–**E**) Heatmaps displaying the expression profiles of genes involved in specific cellular stress response pathways: cytochrome P450-mediated xenobiotic metabolism (**C**), ATP-binding cassette (ABC) transporter activity (**D**), and lysosomal degradation (**E**). Rows represent individual biological replicates from the control (CK) and 120 Gy treatment (R120) groups, while columns represent individual gene symbols (listed at the bottom). The color scale indicates standardized relative expression levels, ranging from blue (low expression, Z-score = −2) to green (high expression, Z-score = 2).

Exposure to the phytosanitary dose of 120 Gy elicited a much more extensive transcriptional response than the sub-effective dose. GO enrichment analysis revealed the perturbation of core biological processes, including cuticle structural components, proteolysis, transmembrane transport, redox homeostasis, and endopeptidase activity (Figure 3A). Notably, many of these pathways showed distinct expression patterns compared to the 15 Gy treatment. KEGG pathway analysis confirmed the disruption of core metabolic pathways overlapping with the 15 Gy response, such as protein digestion and absorption, starch and sucrose metabolism, and tyrosine metabolism (Figure 3B). Furthermore, significant alterations in retinol metabolism, nitrogen metabolism, and glycolysis indicated a systemic disruption of metabolic homeostasis. The enrichment of the non-homologous end joining (NHEJ) pathway suggests substantial genomic damage, as this is a key mechanism for repairing DNA double-strand breaks (Figure 3B). Additionally, the pronounced enrichment of cytochrome P450-mediated xenobiotic metabolism (Figure 3C), ABC transporter activity (Figure 3D), and the lysosomal degradation pathway (Figure 3E) reflected robust cellular stress responses. Collectively, these signatures demonstrate that high-dose X-ray irradiation triggers a multi-system collapse, compromising the biochemical activities and genomic integrity required for larval survival and development.

### 3.4. Comparative Analysis of X-Ray Irradiation-Induced Gene Expression Changes

**Figure 4:**
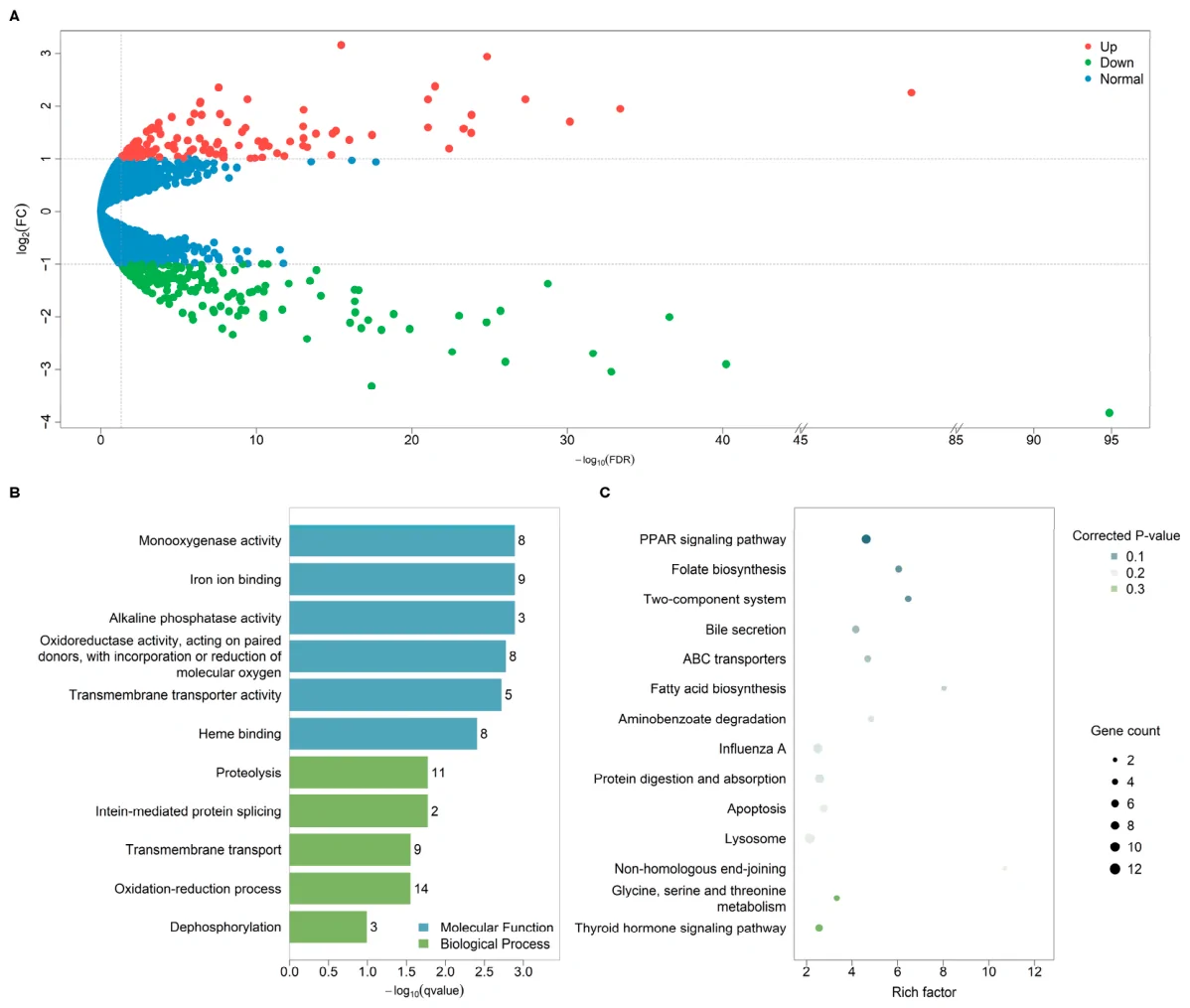
Analysis of dose-dependent transcriptomic responses to irradiation. (**A**) Volcano plot comparing DEGs in larvae treated with 120 Gy versus 15 Gy. Each individual dot represents a single gene. The *x*-axis represents the statistical significance as the negative base-10 logarithm of the false discovery rate (−log_10_ FDR), while the *y*-axis represents the magnitude of expression change as the log_2_ fold change (log_2_FC). Significantly up-regulated genes (meeting the threshold of log_2_FC ≥ 1 and FDR < 0.01) are highlighted in red, and significantly down-regulated genes (log_2_FC ≤ −1 and FDR < 0.01) are highlighted in green. (**B**) Bar chart displaying the top enriched GO terms, categorized into Molecular Function (blue bars) and Biological Process (green bars). The *y*-axis lists the specific enriched terms, while the *x*-axis represents the enrichment significance level (−log_10_ q-value). The numerical value at the end of each bar indicates the exact count of DEGs assigned to that term. (**C**) KEGG pathway enrichment analysis represented as a bubble plot. The *y*-axis lists the affected pathways, and the *x*-axis represents the Rich factor (defined as the proportion of DEGs mapped to a given pathway relative to the total number of annotated genes in that pathway). The size of each bubble corresponds to the total number of DEGs associated with the pathway, and the color intensity represents the corrected *p*-value (false discovery rate), with darker shades indicating higher statistical significance.

To elucidate dose-specific transcriptional responses, we directly compared the gene expression profiles of larvae exposed to 15 Gy versus 120 Gy irradiation. This pairwise contrast identified 277 DEGs (Table S3). Specifically, 172 genes were down-regulated and 105 were up-regulated at 120 Gy (Figure 4A). GO enrichment analysis revealed that the 120 Gy dose significantly perturbed key biological processes, including redox homeostasis, proteolysis, transmembrane transporter activity, and metabolic enzyme function (Figure 4B). KEGG pathway analysis indicated that 120 Gy irradiation further disrupted protein digestion and absorption pathways and impaired core metabolic and biosynthetic processes (Figure 4C). Furthermore, pathways associated with ABC transporter-mediated detoxification, lysosomal degradation, and NHEJ DNA repair were more strongly enriched at 120 Gy (Figure 4C). This enrichment reflects heightened cellular stress and genotoxic damage.

### 3.5. Gene Expression Pattern Clustering Analysis

**Figure 5:**
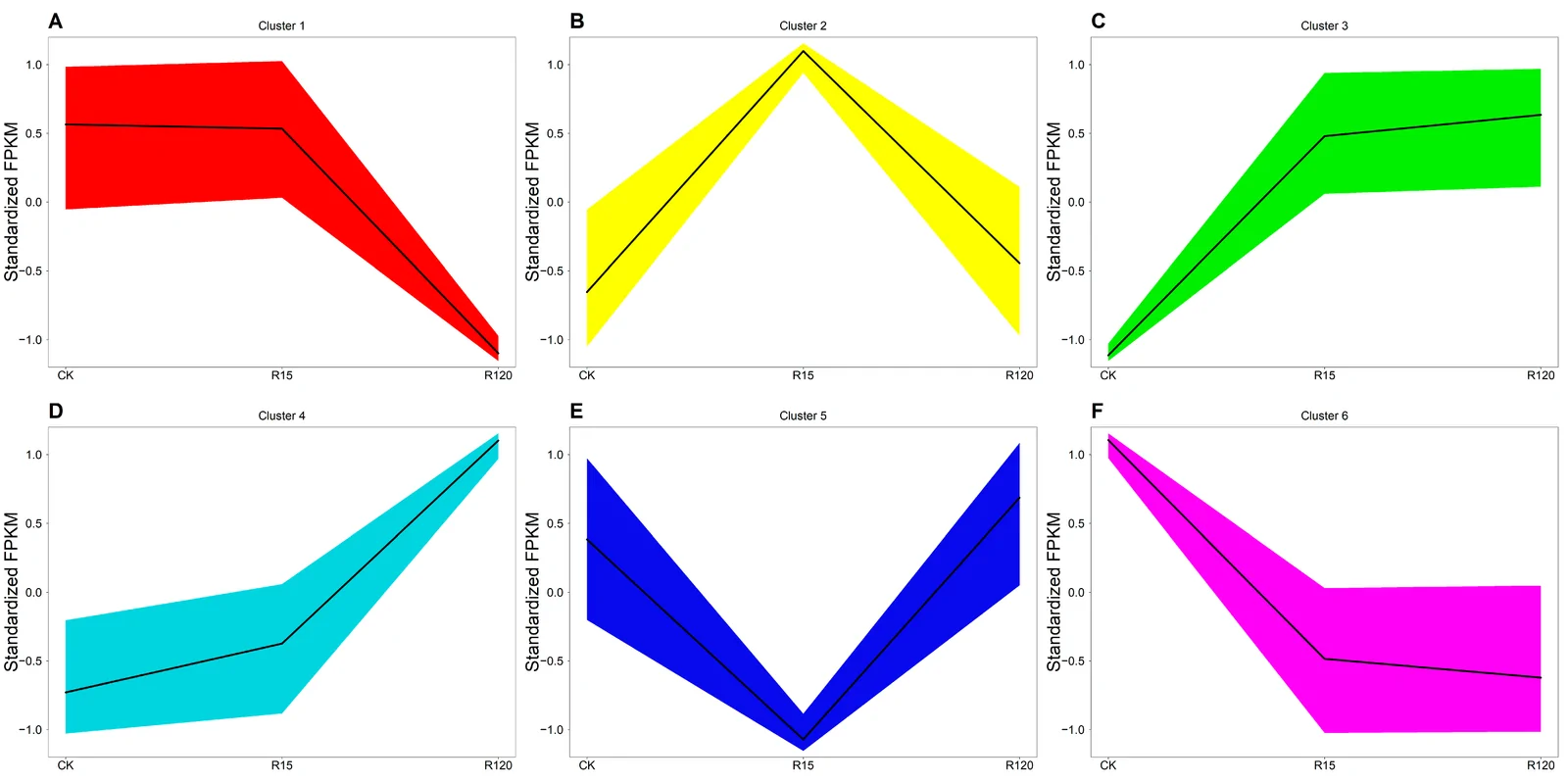
Gene expression pattern clustering analysis. (**A**–**F**) Standardized expression profiles for the six selected gene clusters (Cluster 1–6) identified by K-means clustering. The *y*-axis represents standardized FPKM (Fragments Per Kilobase of transcript per Million mapped reads) values, and the *x*-axis represents the experimental conditions: CK (control, 0 Gy), R15 (15 Gy irradiation), and R120 (120 Gy irradiation). The black line in each panel indicates the mean expression profile (centroid) for each cluster, while the shaded colored area represents the variation or confidence interval of gene expression within the cluster. Cluster 1 shows dose-dependent down-regulation (**A**). Cluster 2 (**B**) and Cluster 3 (**C**) exhibit transient induction at 15 Gy. Cluster 4 displays dose-dependent upregulation (**D**). Cluster 5 shows transient repression at 15 Gy (**E**). Cluster 6 exhibits progressive down-regulation with increasing irradiation dose (**F**). Standardized expression profiles for the six selected gene clusters (Cluster 1–6).

To identify coherent transcriptional modules responding to increasing irradiation doses, we performed K-means clustering analysis and selected six clusters for further study (Figure S2). Genes in Cluster 1 exhibited dose-dependent down-regulation, decreasing progressively as the irradiation dose increased from 0 to 120 Gy (Figure 5). In contrast, genes in Cluster 4 showed dose-dependent up-regulation. These opposing trends likely reflect functionally distinct biological programs. Specifically, Cluster 1 was enriched for genes associated with nutrient metabolism and energy production. Their progressive down-regulation suggests a systematic suppression of core metabolic and bioenergetic pathways (Table S4). Conversely, Cluster 4 was enriched for genes involved in signal transduction, transcriptional regulation, mRNA processing, protein modification, and DNA repair. This signature likely represents a coordinated defense network mobilized to mitigate radiation-induced cellular damage and preserve genomic integrity (Table S4). Genes in the remaining clusters displayed transient or non-linear dynamics, including transient induction (Clusters 2 and 3) and repression (Clusters 5 and 6). Collectively, these expression patterns provide a systemic view of how *B. dorsalis* larvae transcriptionally rewire in response to escalating radiation stress. Furthermore, they highlight specific gene sets as promising biomarkers for irradiation efficacy.

### 3.6. Selection of Candidate Biomarker Genes for Phytosanitary Irradiation

**Figure 6:**
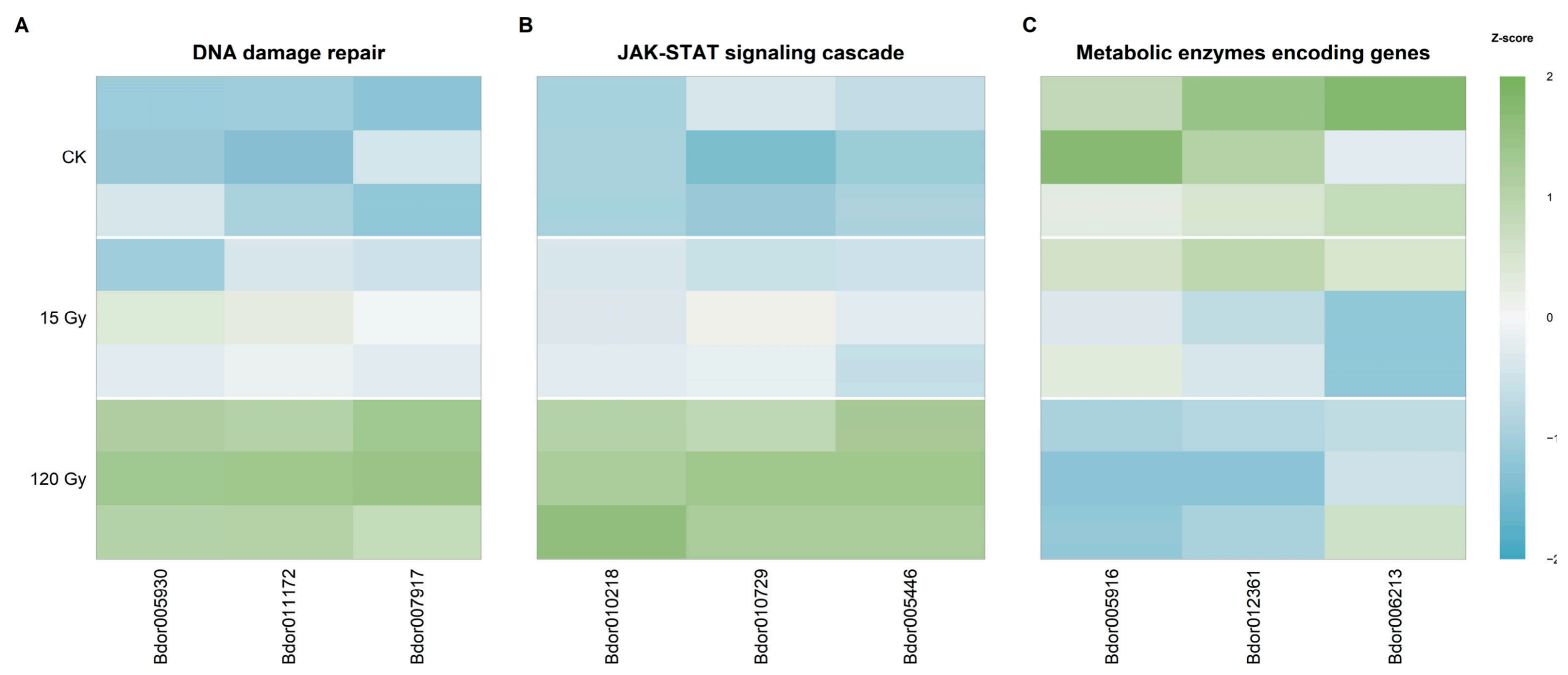
Candidate biomarker genes for irradiation efficacy assessment. Heatmaps display the normalized expression levels (Z-score) of representative genes involved in DNA damage repair (**A**), the JAK-STAT signaling cascade (**B**), and metabolic homeostasis (**C**). Rows represent individual biological replicates from the control (CK), 15 Gy and 120 Gy treatment groups, while columns represent individual gene symbols (listed at the bottom). The color scale indicates standardized relative expression levels, ranging from blue (low expression, Z-score = −2) to green (high expression, Z-score = 2). DNA repair genes and JAK-STAT pathway components show dose-dependent up-regulation, while metabolic enzyme genes exhibit dose-dependent down-regulation.

Based on functional relevance, expression specificity, and dose-dependent responsiveness, we identified nine candidate biomarker genes for validating phytosanitary irradiation in *B. dorsalis* larvae. Three genes involved in DNA damage repair were significantly up-regulated exclusively at the 120 Gy dose, with no detectable induction at 15 Gy (Figure 6A). These included *Bdor000930* (encoding DNA repair endonuclease XPF), *Bdor011172* (encoding X-ray repair cross-complementing protein 5, XRCC5), and *Bdor006799* (encoding DNA repair protein RAD50). Similarly, three core components of the JAK-STAT signaling cascade also exhibited strict dose-dependent up-regulation, responding only to phytosanitary-level irradiation (Figure 6B). These included an unpaired (Upd) ligand encoded by *Bdor010220*, the domeless (Dome) receptor encoded by *Bdor010729*, and the downstream target gene *Myc* (*Bdor000446*). The coordinated upregulation of ligands, receptors, and downstream targets indicates robust pathway activation. Finally, three metabolic enzymes encoding genes were selectively down-regulated at 120 Gy, reflecting dose-specific metabolic suppression (Figure 6C). These included *Bdor006818* (encoding Lysozyme B), *Bdor012361* (encoding Lysozyme C), and *Bdor004213* (encoding Glucose dehydrogenase). The dose-sensitive expression pattern of these nine transcripts positions them as robust molecular indicators for monitoring irradiation efficacy.

## 4. Discussion

The complete inhibition of adult emergence at 120 Gy observed in this study confirms its phytosanitary efficacy. This finding aligns directly with the IPPC’s recommendation of a minimum absorbed dose of 116 Gy to prevent adult emergence in *B. dorsalis* [24]. Given the global significance of *B. dorsalis* as a quarantine pest and the increasing need to replace chemical fumigants, these results strongly support the wider adoption of X-ray irradiation as a phytosanitary treatment [24,25].

While the biological efficacy of ionizing radiation against tephritid fruit flies has been well documented [17,18,19,20,21,22,23], the molecular mechanisms driving dose-dependent mortality remain poorly characterized. By integrating transcriptomic data across X-ray irradiation doses, we delineated a dose-responsive regulatory network in *B. dorsalis* larvae. This network orchestrates cellular stress responses, metabolic disruption, and apoptosis. These findings identify a set of molecular signatures that can be leveraged to verify the efficacy of irradiation-based phytosanitary treatments. Furthermore, our findings also support dose optimization for quarantine applications.

The transcriptional response of *B. dorsalis* larvae to X-ray irradiation exhibited a clear dose-dependent pattern that closely mirrored the observed biological outcomes. At the sub-effective dose (15 Gy), irradiation likely induced metabolic perturbations, oxidative stress, and DNA damage. However, these effects remained below the threshold required to trigger larval mortality. In contrast, exposure to the phytosanitary dose (120 Gy) elicited profound impacts on the larval transcriptome, driving irreversible developmental arrest and robust lethality. Genes commonly dysregulated across both doses were predominantly associated with cuticle structure, protein metabolism, and redox homeostasis. These findings partially recapitulate transcriptomic responses previously identified in other insects, indicating conserved molecular targets of irradiation [27,28,29,30,31].

Higher irradiation doses induced a more pronounced breakdown of essential metabolic and biosynthetic networks. Pathways involving cytochrome P450, ABC transporters, NHEJ DNA repair, and lysosomal degradation were significantly perturbed. The differential expression of P450 and ABC transporter genes likely represents a compensatory response to mitigate radiation-induced oxidative stress and clear toxic byproducts [32,33,34]. Three genes homologous to the DNA repair factors XPF [35], XRCC5 [36], and RAD50 [37] were selected as biomarkers for monitoring irradiation efficacy. Their dose-specific up-regulation indicates that 120 Gy induces massive DNA damage. The prolonged activation of the NHEJ pathway suggests that the cellular repair machinery was overwhelmed by this damage, ultimately driving programmed cell death [38]. The concurrent dysregulation of lysosomal pathways further indicates a loss of cellular homeostasis [39]. Furthermore, the expressions of key metabolic enzymes, including lysozymes [40] and Gld [41], were significantly down-regulated. These observations suggest that cells under high-dose irradiation lose their fundamental capacity for macromolecular turnover and energy metabolism, leading to massive apoptosis.

A striking feature of the dose-specific response was the selective activation of the JAK-STAT signaling pathway at 120 Gy. The JAK-STAT pathway regulates diverse processes, including cell differentiation, tissue homeostasis, apoptosis, immune defense, and stress adaptation [42]. Its core signaling architecture is highly conserved across metazoans. In *D. melanogaster*, the signaling transduction is initiated when cytokine-like Upd ligands bind to the Dome receptor. This triggers JAK-mediated phosphorylation of STAT proteins [43]. Phosphorylated STATs dimerize and translocate to the nucleus to drive target gene expression [44]. In *B. dorsalis*, we observed a highly coordinated, dose-dependent transcriptional induction of this cascade. Two Upd-like ligand genes (*Bdor010218* and *Bdor010220*) were up-regulated following irradiation. Notably, *Bdor010220* was exclusively sensitive to the 120 Gy dose. Concurrently, the *Dome* homolog (*Bdor010729*) and the downstream JAK-STAT target gene *Myc* (*Bdor000446*) were induced only at the phytosanitary dose. This tightly coordinated upregulation of ligands, receptors, and canonical transcriptional targets strongly suggests robust and specific pathway engagement under severe stress. Irradiation has also been shown to activate the JAK-STAT pathway in *D. melanogaster* [45,46,47]. A recent study found that X-ray irradiation of *D. melanogaster* larval wing imaginal discs caused massive DNA damage and cell apoptosis [48]. Single-cell transcriptomic analysis revealed altered expression of genes involved in DNA damage repair, defense against reactive oxygen species, and apoptosis, consistent with our mRNA sequencing results. Notably, irradiation induced the Upd ligands in a spatially heterogeneous manner. Cells in distinct regions of the wing disc up-regulated *Upd* to markedly different extents [48]. It would be of interest to examine whether *Upd* induction in *B. dorsalis* exhibits comparable heterogeneity, both across tissues and among cell populations within a given tissue. Addressing this question would provide deeper insight into the conserved and divergent roles of JAK-STAT signaling in insect radiation responses.

Currently, phytosanitary treatment validation relies heavily on time-consuming biological assays [17,18,19,20,49]. The biomarker genes identified herein could enable the development of rapid diagnostic tools to verify whether absorbed doses meet phytosanitary thresholds. Such biomarkers are highly valuable for quality assurance in commercial facilities, where dose distribution can be heterogeneous due to commodity density, packaging, or positioning. Furthermore, understanding how key stress-response and cellular homeostasis pathways respond to radiation may inform the development of synergistic treatment strategies. For example, combining sub-lethal irradiation with mild thermal or atmospheric stressors could potentially lower the required absorbed dose without compromising quarantine security [7,8,9,26].

While this study provides insights into the transcriptional landscape of irradiated *B. dorsalis* larvae, several avenues warrant further investigation. Functional validation of the identified DEGs is essential to confirm their direct roles in radiation tolerance. This can be achieved through RNA interference or CRISPR-based approaches [50,51,52,53]. Additionally, integrating transcriptomic data with proteomic and metabolomic analyses could reveal the comprehensive regulatory network underlying irradiation response. Future studies should also evaluate dose responses across multiple life stages and varying commodity conditions to refine real-world phytosanitary protocols.

## Data Availability

The original contributions presented in this study are included in the article/Supplementary Material. Further inquiries can be directed to the corresponding authors.

## Supplementary Materials

The following supporting information can be downloaded at: https://www.mdpi.com/article/10.3390/insects17080804/s1, Two supplementary figures and three supplementary tables are published online alongside the manuscript. Figure S1: Sample correlation and hierarchical clustering analysis of transcriptomic profiles in B. dorsalis larvae. Figure S2: Determination of the optimal number of clusters (k). Table S1: List of DEGs induced by 15 Gy exposure. Table S2: List of DEGs induced by 120 Gy exposure. Table S3: List of DEGs identified in the pairwise comparison between the 120 Gy and 15 Gy irradiation treatments. Table S4: List of genes in different clusters.
