# Supplementary material for: A Comparative Transcriptomic Analysis of X-Ray Irradiation Responses Identifies Candidate Biomarker Genes in Bactrocera dorsalis Larvae
Source: Insects. 2026 Aug 3;17(8):804. doi: 10.3390/insects17080804 (PMC13512971; doi:10.3390/insects17080804)
Supplement: Supplementary file 1 [file insects-17-00804-s001.zip › Li et al Supplementary information.pdf]

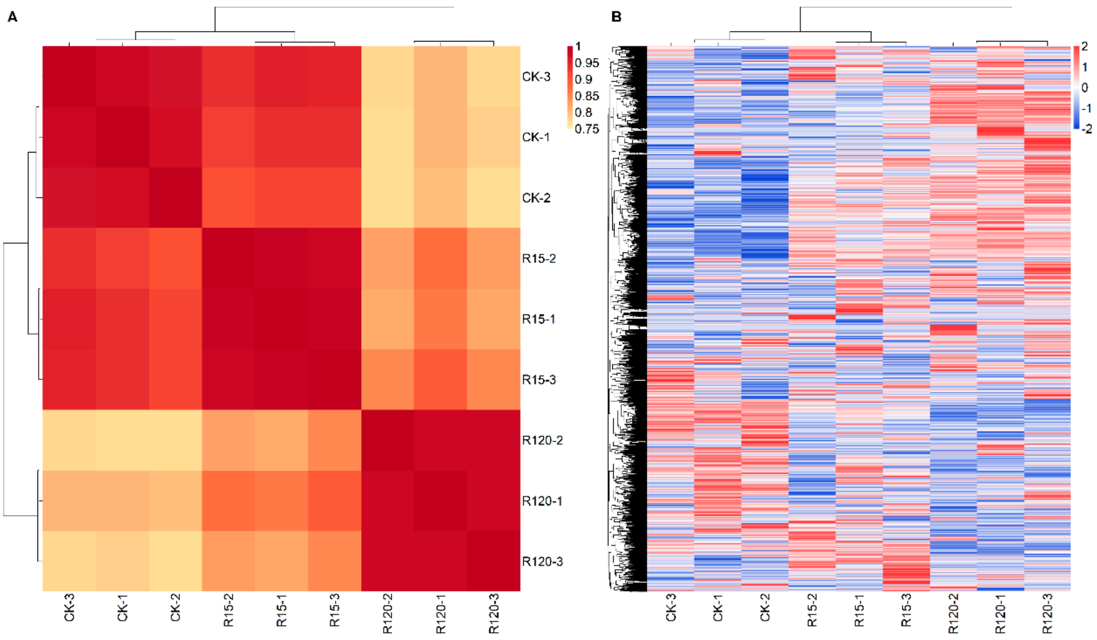

Supplementary Figure S1. Sample correlation and hierarchical clustering analysis of transcriptomic profiles in *B. dorsalis* larvae. (A) Pearson correlation heatmap of all samples based on FPKM values. The color gradient represents the correlation coefficient, ranging from light yellow (lower correlation) to dark red (high correlation, r > 0.90). The tight clustering of biological replicates within each group (CK, R15, and R120) demonstrates the high reproducibility and reliability of the RNA-seq data.

(B) Hierarchical clustering heatmap showing global gene expression patterns across different groups. The color scale indicates standardized expression levels (Z-score), with red representing high expression and blue representing low expression. The dendrogram reveals a clear separation among the control (CK) and irradiation treatment groups (R15 and R120).

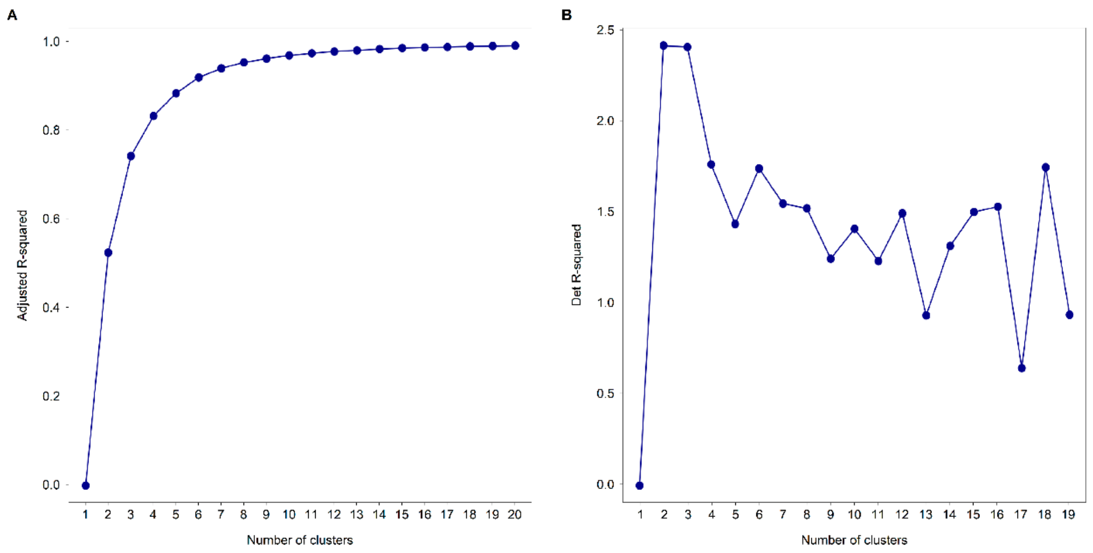

Supplementary Figure S2. Determination of the optimal number of clusters (*k*). The left panel shows the Adjusted R-squared values as a function of the number of clusters, demonstrating a plateau after *k* = 6, which indicates that six clusters are sufficient to capture the variance in gene expression. The right panel shows the difference in R-squared values between successive cluster numbers.
